# Impact of Adaptive Radiation Therapy on Toxicity in Prostate Cancer: A Scoping Review

**DOI:** 10.3390/biomedicines14020370

**Published:** 2026-02-05

**Authors:** Miao Li, Jerry C. F. Ching, Julian T. Tong, Jacky Y. K. Man, Rico H. M. Hung, Vincent W. S. Leung, Curtise K. C. Ng

**Affiliations:** 1 Department of Health Technology and Informatics, Faculty of Health and Social Sciences, The Hong Kong Polytechnic University, Hong Kong SAR, China; miaoo.li@connect.polyu.hk (M.L.); jerrycf.ching@connect.polyu.hk (J.C.F.C.); julian.tong@connect.polyu.hk (J.T.T.); yuikuen.man@connect.polyu.hk (J.Y.K.M.); 2 Department of Clinical Oncology, Pamela Youde Nethersole Eastern Hospital, Hong Kong SAR, China; 3 Curtin School of Diagnostic and Therapeutic Sciences, Curtin University, GPO Box U1987, Perth, WA 6845, Australia; curtise.ng@curtin.edu.au; 4 Curtin Medical Research Institute (Curtin MRI), Faculty of Health Sciences, Curtin University, GPO Box U1987, Perth, WA 6845, Australia

**Keywords:** artificial intelligence, auto-planning, auto-segmentation, computed tomography, conventional fractionated radiotherapy, deep learning, magnetic resonance imaging, organs at risk, stereotactic body radiotherapy, target volume

## Abstract

**Background/Objectives**: Existing literature reviews have not focused on the acute and late toxicities of non-magnetic resonance imaging (MRI)-guided adaptive radiation therapy (ART), compared the impacts of non-MRI-guided versus MRI-guided ART, or evaluated the effectiveness of adaptive conventional fractionated radiation therapy (CFRT) and stereotactic body radiation therapy (SBRT) in relation to toxicity in prostate cancer (PCa). The purpose of this scoping review was to systematically identify original articles and evaluate the impact of ART on toxicity in PCa in a comprehensive manner. **Methods**: A literature search was conducted using electronic databases on 17 June 2025, identifying 27 eligible papers. **Results**: The overall median toxicities of ART in PCa were 15.0% (acute grade 1 gastrointestinal (GI)), 1.0% (acute grade 2 GI), 0.0% (acute grade 3 GI), 47.1% (acute grade 1 genitourinary (GU)), 9.6% (acute grade 2 GU), 0.0% (acute grade 3 GU), 10.0% (late grade 1 GI), 2.0% (late grade 2 GI), 0.0% (late grade 3 GI), 29.7% (late grade 1 GU), 5.0% (late grade 2 GU), and 0.0% (late grade 3 GU). The choice of image guidance modality for ART does not appear to substantially influence toxicity; however, dedicated commercial ART systems may contribute to reducing toxicity to lower levels in PCa. Furthermore, the toxicity rates of adaptive CFRT and SBRT were comparable. **Conclusions**: Adaptive CFRT may be considered when SBRT is unsuitable for certain patients, without increasing the risk of side effects. However, further research is warranted to evaluate dedicated commercial cone-beam computed tomography (CT)- and CT-guided ART systems.

## 1. Introduction

Prostate cancer (PCa) is the fourth most common cancer overall and the second most common cancer in men worldwide [1,2,3]. Localized PCa accounts for approximately 80% of all cases [4,5]. Active surveillance, radical prostatectomy, radiation therapy (RT) alone, and RT combined with androgen deprivation (hormone) therapy are commonly used to treat localized and regional PCa [4,6,7]. According to recent literature reviews and meta-analysis, both radical prostatectomy and RT are equally effective treatments for localized PCa [5,8,9]. However, RT techniques influence treatment outcomes such as clinical effectiveness (e.g., biochemical control) and toxicity [4,6,7]. RT-related toxicities in PCa include gastrointestinal (GI) effects, such as diarrhea and rectal bleeding, as well as genitourinary (GU) toxicities, such as urinary frequency and hematuria [10,11,12,13]. The Radiation Therapy Oncology Group (RTOG) and European Organization for Research and Treatment of Cancer (EORTC) toxicity criteria are widely recognized standards for grading acute (within 3 months of RT) and late toxicities (beyond 3 months) [10,11,12]. The grading scale is defined as follows: 0 (no symptoms), 1 (mild), 2 (moderate), 3 (severe), 4 (life-threatening), and 5 (death) [10].

The concept of adaptive RT (ART), a form of image-guided RT, was first proposed by Yan et al. in 1997 [14,15,16,17]. The essence of ART lies in systematic image-based monitoring of treatment variations—such as setup errors and changes in the size and location of the tumor (target volume (TV)) and adjacent healthy tissues (organs at risk (OARs))—throughout the treatment course, as well as modifications to segmentations of TV and OARs and treatment plans, with the aim of increasing the clinical effectiveness of RT and reducing toxicity [14,15,16,17,18].

ART is particularly important in the treatment of PCa, as OARs, such as the rectum and urinary bladder, are in close proximity to the prostate. Radiation dose escalation for PCa can therefore readily increase GI and GU toxicities [18,19,20]. In the early stage of ART implementation, electronic portal imaging device (EPID) was employed to produce two-dimensional (2D) X-ray images of patients for adjustment [18,21,22,23,24]. However, in recent years, three-dimensional (3D) imaging techniques, such as cone-beam computed tomography (CBCT) [16,24] and magnetic resonance imaging (MRI), have been predominantly used for ART [17,19,25,26]. With the maturation of deep learning (DL)-based automatic segmentation and planning in RT [4,27,28], ART has also transitioned from offline (re-contouring and planning between treatment sessions) to online (adjustments made immediately before the session while the patient remains in the treatment room), including real-time modes (dynamic adjustments during treatment) enabled by DL technology [16,17,19,20,29].

Over the last 15 years, literature reviews focused on ART in PCa have become available [17,18,19,20,21,22,23,24,25,26]. Most of these are narrative reviews [17,18,20,21,23,24,26], covering ART technology developments [17,18,20,21,23], clinical applications [17,18,20,21,23,24], clinical outcomes [21], limitations and challenges [17,18,20,21,23], and MRI-guided ART study design [26]. Others are systematic reviews specifically about MRI-guided ART [19,22,25]. In 2016, McPartlin et al. systematically reviewed changes in the position of the TV and OARs, as well as the role of MRI-guided ART in addressing these changes [22]. Leeman et al. conducted a meta-analysis in 2023 on the acute toxicity associated with dose escalation technique, specifically MRI-guided adaptive stereotactic body RT (SBRT) [19]. In 2025, Szablewska and Roszkowski broadened the scope of the previous systematic review, examining acute and late toxicities, biochemical control, and associated technical parameters of MRI-guided adaptive SBRT [25].

Existing reviews have not focused on the acute and late toxicities of CBCT- and computed tomography (CT)-guided ART, compared the impacts of non-MRI-guided versus MRI-guided ART, or evaluated the effects of adaptive conventional fractionated RT (CFRT) and SBRT on toxicity in PCa [17,18,19,20,21,22,23,24,25,26]. It is noted that CBCT-guided online ART is a more recent technology compared to MRI-guided online ART [19,20]. The strengths of CBCT-guided online ART over MRI-guided online ART include its applicability to a wider range of patients (e.g., those with ferromagnetic implants or claustrophobia), lower equipment costs, and shorter session times [16,20]. Given the intrinsic limitations of systematic reviews and meta-analyses, which often have a narrow focus, as well as the potential subjectivity and bias of narrative reviews, a scoping review can be considered a pragmatic approach to objectively assess the impact of ART on toxicity in a more holistic manner [30,31]. In this way, the gaps identified in existing literature reviews (i.e., the lack of focus on acute and late toxicities of non-MRI-guided ART, comparisons between non-MRI-guided and MRI-guided ART, and the effectiveness of adaptive CFRT and SBRT in relation to toxicity in PCa) can be addressed [17,18,19,20,21,22,23,24,25,26]. The purpose of this scoping review is to systematically identify original studies and answer the question: “What is the impact of ART on toxicity in PCa?”

## 2. Materials and Methods

This scoping review was conducted according to the Preferred Reporting Items for Systematic Reviews and Meta-Analyses Extension for Scoping Reviews (PRISMA-ScR) guidelines. The review was registered with the Open Science Framework (Registration DOI: 10.17605/OSF.IO/U9Y7G). Its main steps included a literature search, article selection, and data extraction and synthesis [32].

### 2.1. Literature Search

Four electronic journal databases, including Excerpta Medica Database (Embase), PubMed, Scopus, and Web of Science, were searched on 17 June 2025 to identify the literature on the impact of ART on toxicity in PCa over the past 25 years. Based on the focus of the review, the following main keywords were used: adaptive radiation therapy, prostate cancer, and their related terms. Appendix A provides the detailed search strategies (Table A1) [22,24]. The publication year range of 2000–2025 was used, as the clinical implementation of ART consistent with current practice began in the 2000s [21].

### 2.2. Article Selection

Articles were independently selected by two reviewers (ML and VWSL) with discussion to resolve any discrepancy [33]. Table 1 illustrates the article selection criteria [4,28].

**Figure 1:**
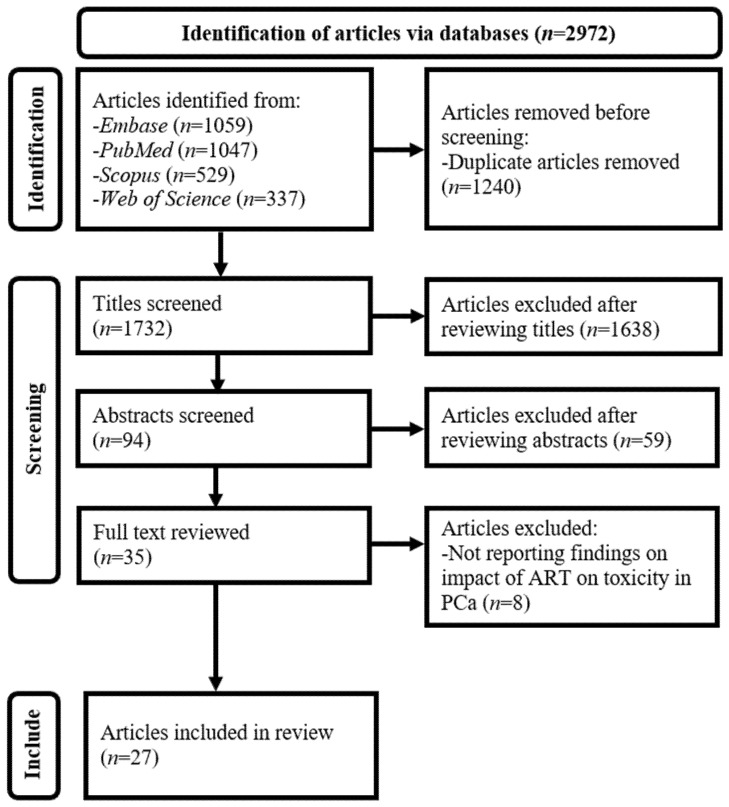
Preferred reporting items for systematic reviews and meta-analyses flow diagram for this scoping review. ART, adaptive radiation therapy; Embase, Excerpta Medica Database; PCa, prostate cancer.

The selection criteria outlined in Table 1 were established in accordance with this review’s purpose [34,35,36]. Details of the article selection process are presented in Figure 1 [32]. The key steps involved removing duplicate records and screening titles, abstracts, and full texts against the predefined criteria. Each unique article identified through the search was retained unless clear grounds for exclusion were determined [34,35,36].

### 2.3. Data Extraction and Synthesis

A data extraction form was developed based on narrative reviews [18,20,21,23,24] and systematic reviews on ART in PCa [19,25]. From each included article, the following data were extracted when available:

1. Bibliographic information: Author and year of publication.
2. Technical parameters: Image guidance modality (e.g., 1.5 T MRI-guided), ART system (such as Elekta Unity, Stockholm, Sweden), fractionation scheme (e.g., SBRT), total dose (such as 35 Gy), fractional dose (e.g., 7 Gy), and planning TV (PTV) coverage (such as prostate gland ± seminal vesicles + 5 mm).
3. Study design and population: Study type (prospective or retrospective), sample size (e.g., 100 patients), tumor, node, and metastasis (TNM) staging of PCa (such as cT1-T2), proportion of patients receiving hormonal therapy, and proportion with rectal spacer or balloon.
4. Toxicity outcomes: Toxicity type classified by timing (e.g., acute, late) and system (such as GI and GU), toxicity grade (e.g., 2, 3), and incidence rate of toxicity [18,19,20,21,23,24,25].

Overall incidence rates of acute and late toxicities were calculated based on the figures for specific side effects when these overall rates were not provided in individual papers. Median incidence rates of acute and late toxicities among the included studies, stratified by grade, were synthesized from those reported [32]. Sensitivity and subgroup analyses of median incidence rates were conducted by removing outlier studies and stratifying by study size (<100 versus ≥100 patients), respectively [37]. The JBI’s critical appraisal tools were used to evaluate the quality of each included article [38].

## 3. Results

Twenty-seven papers met the selection criteria and were included [39,40,41,42,43,44,45,46,47,48,49,50,51,52,53,54,55,56,57,58,59,60,61,62,63,64,65]. Table 2 presents the characteristics of the included studies (study design, population, and technical parameters). More than 80% (22/27) of the articles were MRI-guided ART studies, published from 2019 onward [44,45,46,47,48,49,50,51,52,53,54,55,56,57,58,59,60,61,62,63,64,65]. All but two MRI-guided ART papers were published within the last five years [46,47,48,49,50,51,52,53,54,55,56,57,58,59,60,61,62,63,64,65]. Over 80% (19/22) of these articles investigated the use of MRI-guided ART for SBRT [44,45,46,47,48,49,50,51,52,53,54,55,56,57,59,61,62,63,64], with prospective data collection [44,45,46,47,48,49,51,52,53,56,57,58,59,60,61,62,63,64,65]. Approximately 70% (16/22) used the Elekta Unity ART system [45,46,47,48,51,52,53,54,56,58,59,60,61,62,64,65], while the remainder employed the MRIdian platform (ViewRay Systems, Inc., Oakwood, OH, USA) [44,49,50,55,57,63]. The median sample size across these studies was 60 patients, with a range of 7–425 [44,45,46,47,48,49,50,51,52,53,54,55,56,57,58,59,60,61,62,63,64,65].

Similarly, 80% (4/5) of the CT-guided ART articles recruited patients prospectively [39,40,41,42,43]. However, all but one CT-guided ART article was published more than five years ago (from 2005) and investigated its use for CFRT without the employment of a dedicated ART system [39,40,41,42]. The median sample size across these studies was 280 patients, with a range of 25–962 [39,40,41,42,43], which was greater than that of the MRI-guided ART papers [44,45,46,47,48,49,50,51,52,53,54,55,56,57,58,59,60,61,62,63,64,65]. The article by Goddard et al., published in 2025, was the only exception, employing a dedicated system, Radixact Synchrony (Accuray Incorporated, Madison, WI, USA) for CT-guided ART [43].

Approximately 60% (20/27) of the included studies did not report the proportion of patients treated with a rectal spacer or balloon [39,40,41,42,44,45,47,50,53,54,56,58,60,62,63,64]. Around one-quarter (7/27) did not provide TNM staging information for their PCa patients [41,47,54,55,58,60,64]. However, only three articles (11%) did not report the proportion of patients receiving hormonal therapy [43,47,63]. In addition, the included articles’ quality scores were considerably high (mean: 80.8%, median: 83.3%, and range: 55.6–90.0%) [4,28,39,40,41,42,43,44,45,46,47,48,49,50,51,52,53,54,55,56,57,58,59,60,61,62,63,64,65].

Table 3 presents the median values and ranges of incidence rates for acute and late toxicities of ART, as reported by all included studies [39,40,41,42,43,44,45,46,47,48,49,50,51,52,53,54,55,56,57,58,59,60,61,62,63,64,65], as well as separately for CT-guided [39,40,41,42,43] and MRI-guided ART [44,45,46,47,48,49,50,51,52,53,54,55,56,57,58,59,60,61,62,63,64,65]. The median follow-up time for grading late toxicities across all studies was 10 months, which was well above the minimum threshold of more than three months set by RTOG and EORTC [10,11,12,39,40,41,42,43,44,45,46,47,48,49,50,51,52,53,54,55,56,57,58,59,60,61,62,63,64,65]. Toxicity rates decreased with increasing toxicity grade. The median incidence rates of late toxicities were lower than the corresponding figures for acute toxicities [39,40,41,42,43,44,45,46,47,48,49,50,51,52,53,54,55,56,57,58,59,60,61,62,63,64,65]. Overall, toxicity rates of CT-guided ART [39,40,41,42,43] were comparable to those of MRI-guided ART [44,45,46,47,48,49,50,51,52,53,54,55,56,57,58,59,60,61,62,63,64,65], except that acute grade 2 GI toxicity in CT-guided ART (33.6%), reported by a single study [40], and late grade 1 and 2 GI toxicities [39,40,41,42] appeared notably higher than the corresponding rates in MRI-guided ART [44,45,46,48,49,50,51,52,53,54,55,56,57,58,59,60,61,62,63,64,65]. In addition, Table A2 shows that there were no substantial differences in median toxicity rates across the groups: all studies, without outlier studies, studies with <100 patients, and studies with ≥100 patients [39,40,41,42,43,44,45,46,47,48,49,50,51,52,53,54,55,56,57,58,59,60,61,62,63,64,65].

Table 4 shows the median values and ranges of acute and late toxicity incidence rates for adaptive CFRT and SBRT, based on the included studies. Overall, the two techniques demonstrated similar toxicity rates [39,40,41,42,43,44,45,46,47,48,49,50,51,52,53,54,55,56,57,58,59,60,61,62,63,64,65].

## 4. Discussion

This article is the first scoping review on the impact of ART on acute and late toxicities in PCa, comparing CT-guided versus MRI-guided ART and adaptive CFRT versus SBRT in relation to toxicity, thereby addressing the gaps identified in the previous literature reviews [17,18,19,20,21,22,23,24,25,26]. It covers three dedicated commercial ART systems (Elekta Unity, Radixact Synchrony, and ViewRay MRIdian) and 27 studies [39,40,41,42,43,44,45,46,47,48,49,50,51,52,53,54,55,56,57,58,59,60,61,62,63,64,65]. Hence, it advances the work of Leeman et al.’s 2023 meta-analysis [19] and Szablewska and Roszkowski’s 2025 systematic review [25], which focused on toxicity associated with MRI-guided adaptive SBRT in PCa.

As shown in Table 3, the overall acute toxicities of ART in PCa reported across all included studies were: GI—1.0% (range: 0.0–33.6%) for grade 2 and 0.0% (range: 0.0–4.0%) for grade 3 [40,44,45,46,48,49,50,51,52,53,54,55,57,58,59,60,61,62,63,64,65]; GU—9.6% (range: 0.0–36.0%) for grade 2 and 0.0% (range: 0.0–10.0%) for grade 3 [43,44,45,46,47,48,49,50,51,52,54,55,56,57,58,59,60,61,62,63,64,65]. For MRI-guided ART, the acute toxicities were: GI—1.0% (range: 0.0–17.0%) for grade 2 and 0.0% (range: 0.0–4.0%) for grade 3 [44,45,46,48,49,50,51,52,53,54,55,57,58,59,60,61,62,63,64,65]; GU—9.1% (range: 0.0–36.0%) for grade 2 and 0.0% (range: 0.0–10.0%) for grade 3 [44,45,46,47,48,49,50,51,52,54,55,56,57,58,59,60,61,62,63,64,65]. These findings appear comparable to the pooled estimates and ranges of acute toxicities from Leeman et al.’s 2023 meta-analysis of MRI-guided adaptive SBRT in PCa: acute grade ≥ 2 GI toxicity, 4.0% (range: 0.0–8.3%), and acute grade ≥ 2 GU toxicity, 16.0% (range: 5.0–33.3%). Their pooled estimate of acute grade ≥ 3 GU toxicity was 0.0%, while there was only one case of acute grade ≥ 3 GI toxicity [19]. Given this consistency of findings, our results presented in Table 3 and Table 4 can be considered trustworthy to a certain extent [39,40,41,42,43,44,45,46,47,48,49,50,51,52,53,54,55,56,57,58,59,60,61,62,63,64,65].

Szablewska and Roszkowski’s 2025 systematic review presented toxicity figures only individually from the included papers and did not provide a summary of acute and late toxicities across their 12 MRI-guided adaptive SBRT studies in PCa. This may be attributed to heterogeneity in study designs including interventions and outcome measures among the included MRI-guided adaptive SBRT papers [25]. This is also consistent with Leeman et al.’s analysis, which found significant heterogeneity among their included MRI-guided adaptive SBRT studies in PCa due to variations in approaches to dosimetry, treatment planning, and toxicity management and assessment [19]. Similarly, Table 2 illustrates notable differences in sample sizes, patient characteristics, and planning and treatment techniques, which would explain the wide ranges of incidence rates for certain types and grades of toxicity reported in Table 3 and Table 4 [39,40,41,42,43,44,45,46,47,48,49,50,51,52,53,54,55,56,57,58,59,60,61,62,63,64,65].

Although Table 3 demonstrates that the overall median toxicity rates of CT-guided ART in PCa (0.9% acute grade 3 GI; 56.0% acute grade 1 GU; 12.0% acute grade 2 GU; 0.0% acute grade 3 GU; 2.9% late grade 3 GI; 20.9% late grade 1 GU; 6.0% late grade 2 GU; 4.3% late grade 3 GU) are comparable to those of MRI-guided ART (0.0% acute grade 3 GI; 47.1% acute grade 1 GU; 9.1% acute grade 2 GU; 0.0% acute grade 3 GU; 0.0% late grade 3 GI; 29.7% late grade 1 GU; 4.2% late grade 2 GU; 0.0% late grade 3 GU), the acute grade 2 GI (33.6%) and late grade 1 (26.7%) and 2 GI (17.2%) toxicities of CT-guided ART appear noticeably higher than the corresponding rates for MRI-guided ART (1.0%, 9.1%, and 1.6%, respectively) [39,40,41,42,43,44,45,46,47,48,49,50,51,52,53,54,55,56,57,58,59,60,61,62,63,64,65]. Table 2 shows that the four studies contributing to these findings were published between 2005 and 2018, without the use of any dedicated commercial ART systems. In contrast, Table 3 illustrates that Goddard et al.’s [43] CT-guided adaptive SBRT study, published in 2025 using the Radixact Synchrony platform, reported GU toxicities of 56.0% (grade 1), 12.0% (grade 2), and 0.0% (grade 3), closely matching those of MRI-guided ART (47.1%, 9.1%, and 0.0%, respectively). Furthermore, Goddard et al.’s [43] results were consistent with Leeman et al.’s [19] 2023 meta-analysis, which reported 16.0% for acute grade ≥ 2 GU toxicity and 0.0% for acute grade ≥3 GU toxicity in MRI-guided adaptive SBRT. Taken together, these findings suggest that the choice of image guidance modality may not substantially influence toxicity outcomes in PCa, whereas the use of dedicated commercial ART systems may contribute to reducing toxicity to lower levels due to their online ART capability, which enables adjustment of TV and OAR segmentation, as well as treatment plans that better suit patients’ latest conditions [19,39,40,41,42,43,44,45,46,47,48,49,50,51,52,53,54,55,56,57,58,59,60,61,62,63,64,65]. 

It is well known that MRI provides higher soft-tissue contrast than CT, facilitating delineation of the TV and OARs [4,19,25]. This is crucial for online ART, particularly for real-time ART workflows that require immediate automatic segmentation of these anatomical structures during treatment [4,19,25,66]. In addition, the establishment of the MR-Linac Consortium has helped promote evidence-based practice of MRI-guided ART [67]. Consequently, this has led to a greater number of studies on the use of two DL-empowered, dedicated MRI-guided ART systems: ViewRay MRIdian (released in 2017) and Elekta Unity (available in 2018) (Table 2) [44,45,46,47,48,49,50,51,52,53,54,55,56,57,58,59,60,61,62,63,64,65]. Before these commercial systems became available, only non-MRI-guided ART approaches were feasible [39,40,41,42]. However, MRI-guided ART has several limitations, including higher equipment costs, longer session times, and unsuitability for patients with ferromagnetic implants or claustrophobia [60].

Although promising results for real-time CT-guided ART are noted in Table 3, they are based on a single study involving Radixact Synchrony (available since 2019) [68] by Goddard et al. [43], which retrospectively analyzed a dataset of 25 patients and hence warrants further study. In addition, future research should explore the impact of CBCT-guided online ART, as no study was identified in our literature search despite the availability of the commercial system, Ethos (Varian Medical Systems, Palo Alto, CA, USA) since 2020 [19,20]. This may be attributed to the lowest contrast resolution of CBCT among the three modalities, CBCT, CT, and MRI, and due to the fact that the CBCT-guided online ART system cannot be used for real-time ART [4,16].

Furthermore, SBRT is a more recent technique than CFRT. It enables more precise dose delivery to the TV while reducing exposure to OARs, thereby decreasing toxicity and the number of treatment sessions through the use of higher doses per session, while also improving clinical outcomes [19,25]. However, Table 4 shows that the toxicity rates of adaptive CFRT (17.5% acute grade 1 GI; 1.7% acute grade 2 GI; 0.0% acute grade 3 GI; 56.5% acute grade 1 GU; 2.4% acute grade 2 GU; 0.0% acute grade 3 GU; 10.4% late grade 1 GI; 2.5% late grade 2 GI; 0.0% late grade 3 GI; 28.3% late grade 1 GU; 4.2% late grade 2 GU; 0.0% late grade 3 GU) and SBRT (14.0% acute grade 1 GI; 1.0% acute grade 2 GI; 0.0% acute grade 3 GI; 47.0% acute grade 1 GU; 11.9% acute grade 2 GU; 0.0% acute grade 3 GU; 9.1% late grade 1 GI; 2.0% late grade 2 GI; 0.0% late grade 3 GI; 33.0% late grade 1 GU; 5.9% late grade 2 GU; 0.0% late grade 3 GU) were similar [39,40,41,42,43,44,45,46,47,48,49,50,51,52,53,54,55,56,57,58,59,60,61,62,63,64,65]. This finding is clinically important, given that not all patients with PCa are suitable for SBRT, and adaptive CFRT can be used instead without any increased risk of side effects [54]. However, detailed discussion of technical details of CBCT-, CT-, and MRI-guided ART, including CFRT and SBRT, lies beyond the scope of this review and is available elsewhere [16,19,20,24].

The main limitation of this scoping review is that only papers written in English were included, potentially affecting its comprehensiveness [4,28]. However, it covers 27 articles on ART in PCa, which is greater than the number included in Szablewska and Roszkowski’s 2025 systematic review, which examined only 12 studies on MRI-guided adaptive SBRT in PCa [25].

## 5. Conclusions

This scoping review reveals that the overall median toxicities of ART in PCa were 15.0% (acute grade 1 GI), 1.0% (acute grade 2 GI), 0.0% (acute grade 3 GI), 47.1% (acute grade 1 GU), 9.6% (acute grade 2 GU), 0.0% (acute grade 3 GU), 10.0% (late grade 1 GI), 2.0% (late grade 2 GI), 0.0% (late grade 3 GI), 29.7% (late grade 1 GU), 5.0% (late grade 2 GU), and 0.0% (late grade 3 GU). The choice of image guidance modality for ART does not appear to substantially influence toxicity; however, the use of dedicated commercial ART systems seems to contribute to reducing toxicity to lower levels in PCa. Moreover, the toxicity rates of adaptive CFRT and SBRT were comparable. Therefore, adaptive CFRT may be considered when SBRT is not suitable for particular patients, without increasing the risk of side effects. However, further research is warranted to evaluate dedicated commercial CBCT- and CT-guided ART systems in terms of their impact on toxicity in PCa, as only one study in this area has been identified to date.

In the near future, it is expected that more dedicated commercial CBCT- and CT-guided ART systems will become available, broadening access to ART. In parallel, real-time ART is likely to become increasingly feasible and widely adopted as DL technology continues to advance. These developments collectively represent important directions for the evolution of ART in PCa, with the potential to further reduce toxicity and enhance clinical effectiveness.

## Figures and Tables

**Table 1:** Article selection criteria.

Inclusion Criteria	Exclusion Criteria
Peer-reviewed, original paper Reporting findings on the impact of adaptive radiation therapy on toxicity in prostate cancer Written in English	Brief communications Commentary Conference proceedings Editorials Grey literature Letters Non-peer-reviewed paper (such as those on arXiv platform) Opinion Perspective Review

**Table 2:** Characteristics of studies on impact of adaptive radiation therapy (ART) on toxicity in prostate cancer.

Author and Year	Image Guidance Modality	ART System	Fractionation Scheme	Total Dose (Gy)	Fractional Dose (Gy)	Planning Target Volume Coverage	Study Type	Sample Size (Number of Patients)	Tumor, Node, and Metastasis Staging	Proportion of Patients Receiving Hormonal Therapy	Proportion with Rectal Spacer/Balloon	Article Quality
*Computed Tomography (CT)-Guided*												
Brabbins et al. (2005) [39]	CT-guided	NA	CFRT	70.2–79.2	1.8	Prostate gland +/− SV (for intermediate-risk) + 5 mm (3 mm posteriorly)	Prospective	280	T1b-T3, NX-N0, M0	21.4%	NA	75.0%
Vargas et al. (2005) [40]	CT-guided	NA	CFRT	70.2–79.2	1.8	Prostate gland +/− 1 cm/2 cm of SV	Prospective	331	T1a-T3c, N0, M0	22.0%	NA	77.3%
Park et al. (2012) [41]	CT-guided	NA	CFRT	70.2–79.2	1.8	NA	Retrospective	962	NA	23.7%	NA	72.2%
Hama and Kaji (2018) [42]	CT-guided	NA	CFRT	76.0	2.0	Prostate gland +/− SV + (5 mm–10 mm)	Prospective	26	cT1c-T3a, N0, M0	Neoadjuvant hormone therapy: 76.9%; adjuvant hormone therapy: 42.3%	NA	61.1%
Goddard et al. (2025) [43]	CT-guided	Radixact Synchrony	SBRT	40.0	8.0	Prostate gland +/− SV (for intermediate-risk) + 5 mm (3 mm posteriorly)	Retrospective	25	Stage 1 to 2c	NA	92.0%	55.6%
*Magnetic Resonance Imaging (MRI)-Guided*												
Bruynzeel et al. (2019) [44]	0.35 T MRI-guided	ViewRay MRIdian	SBRT	36.3	7.3	Prostate gland +/− SV (for intermediate-risk) + 5 mm (3 mm posteriorly)	Prospective	101	cT1c-T3b, N0, M0	82.2%	NA	83.3%
Alongi et al. (2020) [45]	1.5 T MRI-guided	Elekta Unity	SBRT	35.0	7.0	Prostate gland +/− SV (for intermediate-risk) + 5 mm (3 mm posteriorly)	Prospective	25	cT1-T2, N0, M0	36.0%	NA	87.5%
Alongi et al. (2021) [46]	1.5 T MRI-guided	Elekta Unity	SBRT	35.0	7.0	Prostate gland +/− SV (for intermediate-risk) + 5 mm (3 mm posteriorly)	Retrospective-prospective	20 (retrospective) and 20 (prospective)	cT1-T2	30.0% (retrospective) and 20.0% (prospective)	100.0% (retrospective) and 0.0% (prospective)	90.0%
de Mol van Otterloo et al. (2021) [47]	1.5 T MRI-guided	Elekta Unity	CFRT and SBRT	60.0 (CFRT) and 36.3/38.6 (SBRT)	3.0 (CFRT) and 7.3/7.7 (SBRT)	NA	Prospective	223	NA	NA	NA	87.5%
Poon et al. (2021) [48]	1.5 T MR-guided	Elekta Unity	SBRT	36.3/40.0	7.3/8.0	Prostate gland +/− SV (for intermediate-/high-risk) + 5 mm (3 mm posteriorly)	Prospective	51	T1 (7.8%), T2 (74.5%), T3 (17.6%), N0, M0	58.8%	19.6%	80.0%
Tetar et al. (2021) [49]	0.35 T MR-guided	ViewRay MRIdian	SBRT	36.3	7.3	Prostate gland +/− SV (for intermediate-/high-risk) + 3 mm	Prospective	101	T1–3b, N0, M0	83.2%	0.0%	87.5%
Ugurluer et al. (2021) [50]	0.35 T MR-guided	ViewRay MRIdian	SBRT	36.3	7.3	Prostate gland +/− SV (for intermediate-/high-risk) + 3 mm	Retrospective	50	T1 (60.0%), T2 (36.0%), T3 (4.0%), N0, M0	28.0%	NA	87.5%
Alongi et al. (2022) [51]	1.5 T MR-guided	Elekta Unity	SBRT	35.0 (low-risk/M1); 36.3 (intermediate-/high-risk)	7.0 (low-risk/M1); 7.3 (intermediate-/high-risk)	Prostate gland +/− SV (for M1, intermediate- and high-risk) + 5 mm (3 mm posteriorly)	Prospective	100	cT1-cT3b, N0, M0	32.0%	37.0%	87.5%
Poon et al. (2022) [52]	1.5 T MR-guided	Elekta Unity	SBRT	40.0	8.0	Prostate gland +/− base of SV (for cT3b, entire SV) + 5 mm (3 mm posteriorly)	Prospective	42	cT2a-T3b, N0, M0	100.0%	31.0%	70.0%
Teunissen et al. (2022) [53]	MR-guided	Elekta Unity	CFRT and SBRT	62.0 (CFRT) and 36.3 (SBRT)	3.1 (CFRT) and 7.3 (SBRT)	NA	Prospective	293	cT1 (51.4%), cT2 (43.8%), cT3 (4.5%), cT4 (0.3%)	13.7%	NA	80.0%
Turkkan et al. (2022) [54]	1.5 T MR-guided	Elekta Unity	CFRT, MHFRT, and SBRT	66.0–78.0 (CFRT), 70.0 (MHFRT), and 36.3 (SBRT)	1.8–2.0 (CFRT), 2.5 (MHFRT), and 7.3 (SBRT)	NA	Retrospective	14	NA	71.0%	NA	88.9%
Gelbart Pridan et al. (2023) [55]	0.35 T MR-guided	ViewRay MRIdian	SBRT	36.3	7.3	Prostate gland + 3 mm	Retrospective	200	NA	28.0%	0.0%	70.0%
Teunissen et al. (2023) [56]	1.5 T MR-guided	Elekta Unity	SBRT	36.3	7.3	Prostate gland +/− SV + 5 mm (for 2 patients, 3 mm posteriorly)	Prospective	425	cT1 (39.2%), cT2 (55.7%), cT3 (5.1%)	18.4%	NA	85.0%
Fink et al. (2024) [57]	0.35 T MR-guided	ViewRay MRIdian	SBRT	37.5	7.5	Prostate gland +/− SV (for intermediate-/high-risk) + 3 mm	Prospective	69	<T3a, N0, M0	12.0%	0.0%	88.9%
Hassan et al. (2024) [58]	1.5 T MR-guided	Elekta Unity	CFRT	70.0–72.0	3.0	NA	Prospective	7	NA	0.0%	NA	87.5%
Poon et al. (2024) [59]	1.5 T MR-guided	Elekta Unity	SBRT	36.3/40.0	7.3/8.0	NA	Prospective	34	T2 (76.5%); T3 (23.5%), N0, M0	76.5%	100.0%	77.3%
Sritharan et al. (2024) [60]	1.5 T MR-guided	Elekta Unity	CFRT	60.0	3.0	Prostate gland +/− SV + 5 mm (3 mm/0 mm posteriorly)	Prospective	140	NA	58.0%	NA	70.0%
Westley et al. (2024) [61]	1.5 T MR-guided	Elekta Unity	SBRT	24.0 (2-fraction) and 36.3 (5-fraction)	12.0 (2-fraction) and 7.3 (5-fraction)	Prostate gland +/− 1 cm/2 cm of SV + 3 mm	Prospective	20	T2 (70%), T3a (30%) (2-fraction) and T2 (80%), T3a (20%) (5-fraction)	100.0%	0.0%	90.0%
Cooper et al. (2025) [62]	1.5 T MR-guided	Elekta Unity	SBRT	24.0 (2-fraction) and 36.3 (5-fraction)	12.0 (2-fraction) and 7.3 (5-fraction)	Prostate gland +/− 2 cm of SV + 3 mm (2-fraction) and prostate gland +/− 1 cm of SV + 3 mm (5-fraction)	Prospective	23 (2-fraction) and 24 (5-fraction)	cT1-T3a (2-fraction) and cT1c-T3a (5-fraction)	100.0%	NA	90.0%
Joye et al. (2025) [63]	0.35 T MR-guided	ViewRay MRIdian	SBRT	36.3/37.5	7.3/7.5	NA	Prospective	209	cT1a-T3b, N0, M0	NA	NA	80.0%
Lalmahomed et al. (2025) [64]	1.5 T MR-guided	Elekta Unity	SBRT	36.3	7.3	Prostate gland +/− SV + 2–3 mm (tight margin group) and prostate gland +/− SV + 5 mm (standard margin group)	Prospective	106 (tight margin group) and 106 (standard margin group)	NA	5.0% (tight margin group) and 6.0% (standard margin group)	NA	81.8%
Poon et al. (2025) [65]	1.5 T MR-guided	Elekta Unity	CFRT	70.0	2.0	NA	Prospective	30	pT2b-pT4, pNX-pN0, M0	96.7%	0.0%	88.9%

CFRT, conventional fractionated radiation therapy; MHFRT, moderate hypofractionated radiation therapy; NA, not available; SBRT, stereotactic body radiation therapy; SV, seminal vesicle.

**Table 3:** Incidence rates for acute and late toxicities of adaptive radiation therapy (ART), computed tomography (CT)-, and magnetic resonance imaging (MRI)-guided ART.

Toxicity			ART		CT-Guided ART		MRI-Guided ART	
Timing	System	Grade	Median Incidence Rate (Range)	Study Reference	Median Incidence Rate (Range)	Study Reference	Median Incidence Rate (Range)	Study Reference
Acute	GI	1	15.0% (0.0–100.0%)	[44,45,46,48,49,50,51,52,53,54,55,57,58,59,60,61,62,63,64,65]	NA	NA	13.6% (0.0–100.0%)	[44,45,46,48,49,50,51,52,53,54,55,57,58,59,60,61,62,63,64,65]
		2	1.0% (0.0–33.6%)	[40,44,45,46,48,49,50,51,52,53,54,55,57,58,59,60,61,62,63,64,65]	33.6% ^1^	[40]	1.0% (0.0–17.0%)	[44,45,46,48,49,50,51,52,53,54,55,57,58,59,60,61,62,63,64,65]
		3	0.0% (0.0–4.0%)	[40,44,45,46,48,49,50,51,52,53,54,55,57,58,59,60,61,62,63,64,65]	0.9% ^1^	[40]	0.0% (0.0–4.0%)	[44,45,46,48,49,50,51,52,53,54,55,57,58,59,60,61,62,63,64,65]
	GU	1	47.1% (1.0–100.0%)	[43,44,45,46,47,48,49,50,51,52,54,55,56,57,58,59,60,61,62,63,64,65]	56.0% ^1^	[43]	47.1% (1.0–100.0%)	[44,45,46,47,48,49,50,51,52,54,55,56,57,58,59,60,61,62,63,64,65]
		2	9.6% (0.0–36.0%)	[43,44,45,46,47,48,49,50,51,52,54,55,56,57,58,59,60,61,62,63,64,65]	12.0% ^1^	[43]	9.1% (0.0–36.0%)	[44,45,46,47,48,49,50,51,52,54,55,56,57,58,59,60,61,62,63,64,65]
		3	0.0% (0.0–10.0%)	[43,44,45,46,47,48,49,50,51,52,54,55,56,57,58,59,60,61,62,63,64,65]	0.0% ^1^	[43]	0.0% (0.0–10.0%)	[44,45,46,47,48,49,50,51,52,54,55,56,57,58,59,60,61,62,63,64,65]
Late	GI	1	10.0% (0.0–43.0%)	[39,40,41,42,49,50,51,52,56,58,60,62,65]	26.7% (15.4–38.0%)	[39,42]	9.1% (0.0–43.0%)	[49,50,51,52,56,58,60,62,65]
		2	2.0% (0.0–26.0%)	[39,40,41,42,49,50,51,52,56,58,60,62,65]	17.2% (3.8–26.0%)	[39,40,41,42]	1.6% (0.0–22.0%)	[49,50,51,52,56,58,60,62,65]
		3	0.0% (0.0–10.0%)	[39,40,41,42,49,50,51,52,56,58,60,62,65]	2.9% (0.0–10.0%)	[39,40,41,42]	0.0% (0.0–3.0%)	[49,50,51,52,56,58,60,62,65]
	GU	1	29.7% (3.8–70.0%)	[39,42,49,50,51,52,56,60,62,65]	20.9% (3.8–38.0%)	[39,42]	29.7% (13.0–70.0%)	[49,50,51,52,56,60,62,65]
		2	5.0% (0.0–15.5%)	[39,41,42,49,50,51,52,56,60,62,65]	6.0% (0.0–15.5%)	[39,41,42]	4.2% (0.0–14.0%)	[49,50,51,52,56,60,62,65]
		3	0.0% (0.0–9.0%)	[39,41,42,49,50,51,52,56,60,62,65]	4.3% (0.0–9.0%)	[39,41,42]	0.0% (0.0–3.0%)	[49,50,51,52,56,60,62,65]

^1^ Median and range are unavailable as a result of only one study reporting toxicity figure. GI, gastrointestinal; GU, genitourinary; NA, not available.

**Table 4:** Incidence rates for acute and late toxicities of adaptive conventional fractionated radiation therapy (CFRT) and stereotactic body radiation therapy (SBRT).

Toxicity			Adaptive CFRT		Adaptive SBRT	
Timing	System	Grade	Median Incidence Rate (Range)	Study Reference	Median Incidence Rate (Range)	Study Reference
Acute	GI	1	17.5% (0.0–46.7%)	[53,58,60,65]	14.0% (0.0–100.0%)	[44,45,46,48,49,50,51,52,54,55,57,59,61,62,63,64]
		2	1.7% (0.0–14.3%)	[53,58,60,65]	1.0% (0.0–33.6%)	[44,45,46,48,49,50,51,52,54,55,57,59,61,62,63,64]
		3	0.0% (0.0%)	[53,58,60,65]	0.0% (0.0–4.0%)	[44,45,46,48,49,50,51,52,54,55,57,59,61,62,63,64]
	GU	1	56.5% (14.3–73.3%)	[56,58,60,65]	47.0% (1.0–100.0%)	[43,44,45,46,47,48,49,50,51,52,54,55,57,59,61,62,63,64]
		2	2.4% (0.0–7.0%)	[56,58,60,65]	11.9% (0.0–36.0%)	[43,44,45,46,47,48,49,50,51,52,54,55,57,59,61,62,63,64]
		3	0.0% (0.0%)	[56,58,60,65]	0.0% (0.0–10.0%)	[43,44,45,46,47,48,49,50,51,52,54,55,57,59,61,62,63,64]
Late	GI	1	10.4% (5.0–38.0%)	[39,40,41,42,56,58,60,65]	9.1% (0.0–43.0%)	[49,50,51,52,62]
		2	2.5% (0.0–26.0%)	[39,40,41,42,56,58,60,65]	2.0% (0.0–22.0%)	[49,50,51,52,62]
		3	0.0% (0.0–10.0%)	[39,40,41,42,56,58,60,65]	0.0% (0.0–3.0%)	[49,50,51,52,62]
	GU	1	28.3% (3.8–63.3%)	[39,41,42,56,60,65]	33.0% (13.0–70.0%)	[49,50,51,52,62]
		2	4.2% (0.0–15.5%)	[39,41,42,56,60,65]	5.9% (0.0–14.0%)	[49,50,51,52,62]
		3	0.0% (0.0–9.0%)	[39,41,42,56,60,65]	0.0% (0.0–3.0%)	[49,50,51,52,62]

GI, gastrointestinal; GU, genitourinary.

## Data Availability

No new data were analyzed in this study. Data sharing is not applicable to this article.

## Abbreviations

The following abbreviations are used in this manuscript:

2D	Two-dimensional
3D	Three-dimensional
ART	Adaptive radiation therapy
CBCT	Cone-beam computed tomography
CFRT	Conventional fractionated radiation therapy
CT	Computed tomography
DL	Deep learning
Embase	Excerpta Medica Database
EORTC	European Organization for Research and Treatment of Cancer
EPID	Electronic portal imaging device
GI	Gastrointestinal
GU	Genitourinary
MHFRT	Moderate hypofractionated radiation therapy
MRI	Magnetic resonance imaging
NA	Not available
OARs	Organs at risk
PCa	Prostate cancer
PRISMA-ScR	Preferred Reporting Items for Systematic Reviews and Meta-Analyses Extension for Scoping Reviews
RT	Radiation therapy
RTOG	Radiation Therapy Oncology Group
SBRT	Stereotactic body radiation therapy
SV	Seminal vesicle
TNM	Tumor, node, and metastasis
TV	Target volume

## Appendix A. Search Strategies Employed for Electronic Scholarly Publication Databases

**Table A1:** Search statements used.

Database	Search Statement	Articles Identified (*n*)
Excerpta Medica Database (Embase)	(‘prostate cancer’/exp OR ‘prostate tumor’/exp OR ‘prostate cancer’ OR ‘prostate carcinoma’) AND (‘adaptive radiotherapy’/exp OR “adaptive radiotherapy” OR “adaptive radiation therapy” OR ‘adaptive planning’ OR ‘treatment adaptation’ OR ‘online adaptive radiotherapy’ OR ‘offline adaptive radiotherapy’ OR ‘MR guided radiotherapy’ OR ‘MRI guided radiotherapy’ OR ‘MR linac’ OR ‘MRIdian’ OR ‘Unity’ OR ‘CBCT guided’ OR ‘cone beam CT guided’) AND (‘radiotherapy’/exp) AND [english]/lim AND [2000–2025]/py	1059
PubMed	(“Prostatic Neoplasms”[Mesh] OR “prostate cancer” OR “prostate carcinoma”) AND (“adaptive radiotherapy” OR “adaptive radiation therapy” OR “ART plan” OR “adaptive planning” OR “online adaptive” OR “offline adaptive” OR “replanning” OR “MR-guided” OR “MRI-guided” OR “MR linac” OR “MR-Linac” OR “magnetic resonance-guided” OR MRgRT OR “CBCT-guided” OR “cone-beam CT” OR “cone beam CT”) AND (radiotherapy OR “radiation therapy”) AND (English[lang]) AND (“2000/01/01”[PDAT]: “2025/12/31”[PDAT])	1047
Scopus	TITLE-ABS-KEY((prostate OR prostatic) W/2 (cancer OR carcinoma OR tumor OR tumour) AND (adaptive W/3 (radiotherapy OR “radiation therapy”) OR “adaptive radiotherapy” OR “online adaptive radiotherapy” OR “MR-guided” W/2 radiotherapy OR “MRI-guided radiotherapy” OR “MR linac” OR “CBCT-guided radiotherapy”)) AND PUBYEAR AFT 1999 AND (LIMIT-TO(LANGUAGE, “English”))	529
Web of Science	TS = ((prostate OR prostatic) NEAR/2 (cancer OR carcinoma OR tumor OR tumour) AND (adaptive NEAR/3 (radiotherapy OR “radiation therapy”) OR “adaptive radiotherapy” OR “adaptive radiation therapy” OR “online adaptive radiotherapy” OR “MR-guided radiotherapy” OR “MRI-guided radiotherapy” OR “MR Linac” OR “CBCT-guided radiotherapy”)) AND LANGUAGE: (English) AND DOCUMENT TYPES: (Article OR Trial) AND Timespan: 2000–2025	337

## Appendix B. Sensitivity and Subgroup Analysis Results for Median Incidence Rates of Acute and Late Toxicities in Adaptive Radiation Therapy (ART)

**Table A2:** Median incidence rates of acute and late gastrointestinal (GI) and genitourinary (GU) toxicities in ART with and without outlier studies and stratified by study size (<100 vs ≥100 patients) [39,40,41,42,43,44,45,46,47,48,49,50,51,52,53,54,55,56,57,58,59,60,61,62,63,64,65].

Toxicity			All Studies	Without Outlier Studies	Studies with <100 Patients	Studies with ≥100 Patients
Timing	System	Grade				
Acute	GI	1	15.0%	13.5%	13.5%	20.1%
		2	1.0%	1.0%	2.9%	2.0%
		3	0.0%	0.0%	0.0%	0.0%
	GU	1	47.1%	47.1%	47.1%	47.0%
		2	9.6%	7.7%	8.3%	11.0%
		3	0.0%	0.0%	0.0%	0.0%
Late	GI	1	10.0%	9.6%	9.6%	10.0%
		2	2.0%	2.0%	3.6%	2.0%
		3	0.0%	0.0%	0.0%	1.0%
	GU	1	29.7%	29.7%	28.6%	30.0%
		2	5.0%	3.2%	0.0%	6.8%
		3	0.0%	0.0%	0.0%	1.0%
